# Rectal specimen self-collection for chlamydia and gonorrhea screening: a cross-sectional feasibility study at a community health center

**DOI:** 10.1186/s40814-021-00928-7

**Published:** 2021-11-15

**Authors:** Marwan S. Haddad, Lauren Bifulco, Jeannie McIntosh, Meghan Mc Clain Garcia

**Affiliations:** 1 Center for Key Populations, Community Health Center, Inc, 675 Main St, Middletown, CT 06457 USA; 2 Weitzman Institute, 19 Grand St, Middletown, CT 06457 USA

**Keywords:** Sexual and gender minorities, Gonorrhea, Chlamydia, STI rectal self-collection, Community health centers, Federally qualified health center (FQHC), Primary care, General practice

## Abstract

**Background:**

Men who have sex with men (MSM) are at increased risk for extra-genital sexually transmitted infections (STIs). Without extra-genital screening, many chlamydia and gonorrhea infections would be missed among MSM. Yet, many barriers exist to extra-genital testing, and, in particular, to rectal collection. Self-collection increases screening and detection of asymptomatic chlamydia and gonorrhea among at-risk MSM and transgender women. This feasibility study assessed use of rectal self-collection and its acceptance among patients and primary care providers (PCPs) at a large, general practice community health center. The primary objective of this project was to assess the feasibility of including rectal self-collection as part of an implementation study looking to embed an STI care program in a safety-net primary care setting that would shift routine screening tasks to non-provider clinical team members such as medical assistants and nurses.

**Methods:**

Three PCPs identified and offered rectal self-collection to their MSM and transgender female patients who were due for routine or risk-based STI screening. For those patients who elected to participate in the study, the PCP’s medical assistant (MA) reviewed the self-collection instructions with them as part of their routine preventive care duties, and patients collected their own sample. Patients and PCPs completed brief cross-sectional surveys assessing the self-collection process.

**Results:**

Of 1191 patients with sexual orientation and gender identity (SOGI) data on file who were seen for a medical visit by one of the three PCPs, 87 (7.3%) identified as MSM or transgender female. Seventy-five were due for rectal screening, of whom 33 (44%) were offered and completed rectal self-collection. Survey results indicated that self-collection was acceptable to and preferred over clinician-collection by both PCPs and patients.

**Conclusions:**

This study demonstrated that rectal self-collection is feasible as part of STI screening in a high-volume primary care setting, and can be administered as part of the clinical tasks that MAs routinely conduct. The overall acceptance by both PCPs and patients will allow the inclusion of rectal self-collection in an implementation study looking to increase STI screening at a large community health center by facilitating MA-led collection during medical provider visits and by establishing standalone nurse-led STI visits.

## Introduction

### Background and objectives

Sexually transmitted infection (STI) rates continue to rise in the USA, with 1.8 million cases of *Chlamydia trachomatis* (chlamydia) and 616,000 cases of *Neisseria gonorrhoeae* (gonorrhea) reported in 2019 [1]. Those at increased risk include racial and ethnic minorities and men who have sex with men (MSM) [1, 2]; the burden of STIs is further increased for medically underserved and vulnerable patients [3]. A 2018 survey of 326 local health departments in the USA determined that over one-third of service areas had no clinics that offered STI screening, diagnosis, and treatment services for patients in the healthcare safety net [4]. Safety-net treatment providers such as federally qualified health centers (FQHCs) allow access to screening, diagnosis, and treatment regardless of insurance status or ability to pay, and provide general primary care services to more than 30 million Americans at over 13,000 delivery sites [5], presenting an opportunity to fill STI service coverage gaps.

The United States STI National Strategic Plan 2021‑2025 identifies MSM as a priority population for STI risk reduction and care improvement [2]. Though chlamydia and gonorrhea testing in MSM has predominantly focused on urethral detection, MSM are also at increased risk for extra-genital (pharyngeal and rectal) STIs, based on exposure through oral and anal sex. Extra-genital chlamydia and gonorrhea may be present without concurrent urogenital infection [6] and are frequently asymptomatic among MSM [7, 8], which increases risk of transmission to sexual partners [9, 10] and perpetuates reservoirs of infection [9, 11]. Given estimated mean prevalence rates among MSM of approximately 9% and 5% for rectal chlamydia and gonorrhea, respectively [8, 12], routine rectal screening is indicated at least annually for asymptomatic MSM who engage in receptive anal sex, with more frequent screening indicated for those who engage in ongoing at-risk sexual behaviors and/or have multiple partners or partners who have other sexual partners [13]. Screening recommendations for transgender women, who may engage in some of the same sexual practices as MSM, are currently based on individual sexual behavior [13]. Though studies of STI prevalence among transgender women have been limited in number and scope and have primarily focused on urogenital detection, a 2020 meta-analysis found estimated STI prevalence rates in transgender women ranging from 2.1‑19.1% for gonorrhea and 2.7‑24.7% for chlamydia [14].

Barriers to chlamydia and gonorrhea rectal screening include increased visit complexity and time compared to urogenital testing; lack of provider awareness regarding need for extra-genital screening; stigma experienced or perceived by patients and/or providers related to sexual behavior; and discomfort with taking or providing a detailed sexual risk assessment and with the collection of rectal samples [7]. Self-collection of extra-genital samples for STI screening is one potential method of overcoming these barriers. Self-collection has been shown to identify asymptomatic infection [8, 15], to be equally as or more effective than clinician-collection [16], and to be preferred by patients [17, 18]. Self-collection of rectal samples may increase screening rates for at-risk MSM [19] by reducing stigma, improving patient comfort [20], and reducing demand on healthcare provider time [8].

To-date, the use of rectal self-collection as part of routine STI screening for MSM and transgender women has not been studied at a large, high-volume general population community health center. Challenges to feasibility in this setting include identifying patients at increased risk who would benefit from routine screening; patient discomfort and acceptability of rectal collection given the sensitive and invasive nature of the test; and PCP time, effort, and uneasiness with sexual health-related assessments.

This study was conducted among asymptomatic adult MSM and transgender female patients of three primary care providers (PCPs) at Community Health Center, Inc. (CHCI), a large, multi-site FQHC in Connecticut. The primary objective of this project was to assess the feasibility of using rectal self-collection for STI screening in MSM and transgender women during a primary care medical visit in a busy community health center setting. If the primary feasibility outcomes are met, rectal self-collection will be incorporated as an essential component of an implementation study that would examine shifting routine STI screening tasks to non-provider clinical team members such as medical assistants (MAs) and nurses. The secondary objective was to develop an understanding of whether having sexual orientation and gender identity (SOGI) information in the chart helped PCPs offer rectal self-collection to those MSM and transgender women who required screening.

We hypothesized that we would be able to develop a process to identify patients at increased risk who would benefit from routine screening, and that self-collection would be easy to adopt and would be accepted and preferred by the majority of patients and PCPs.

## Methods

### Trial design

Cross-sectional, observational feasibility study.

### Participants

Our study enrolled MSM and transgender female patients identified by the PCP as requiring rectal chlamydia and gonorrhea screening during a primary care medical visit. Those offered participation had a visit during the study period, were 18+, English-speaking, reported history of receptive anal sex, and were due for routine or risk-based rectal STI testing. Patients were recruited from the panels of three PCPs practicing at three sites of a multi-site FQHC. This study followed the Consolidated Standards of Reporting Trials (CONSORT) statement extension to pilot trials reporting guideline [21].

### Interventions

PCPs identified eligible patients due for rectal STI screening during medical visits based on their review of SOGI data from the electronic health record (EHR), sexual risk assessments, and recent STI testing. Patients agreeing to rectal STI screening were offered participation in the rectal self-collection study or standard of care which would require the patient to undress, be draped, and have a chaperone present with the PCP for the intimate examination and specimen collection. Patients who opted to participate in the study were consented by the PCP or MA toward the end of the medical visit, verbally instructed on how to self-collect, given the opportunity to ask questions, and provided with a set of printed instructions. MAs provided the instruction and received the self-collected specimen, which allowed the PCP to wrap up the medical visit as per their typical workflow and move on to the next scheduled patient. Patients were then left to self-collect their specimen in private in the exam room or bathroom. Patients and PCPs completed a short survey after self-collection or at the end of the study, respectively.

### Outcomes

For our primary objective, the feasibility of implementing rectal self-collection was contingent on PCPs’ and patients’ acceptability of the rectal self-collection process. We used cross-sectional self-report surveys to measure acceptability based on the following primary outcomes: PCPs’ time and effort; PCPs’ likelihood of rectal screening if self-collection was offered; and PCPs’ preference for self-collection vs. clinician-collection; patients’ ability to self-collect a specimen; patients’ physical comfort with self-collection; and patients’ preference for self-collection versus clinician-collection. We also captured qualitative data on the types of questions patients asked regarding self-collection, and operational data on number of self-collected specimens that were acceptable for laboratory analyses.

For our secondary objective, we wanted to understand whether having SOGI information in the chart helped the PCPs offer rectal self-collection to those MSM and transgender women who required screening. We used the following measures to assess our secondary outcomes: total number of patients seen by the PCPs for medical visits; total number of those patients who had SOGI information in the chart; of those, the number of people who identified as MSM or transgender female; the proportion of those patients who were due for rectal screening; and the proportion of those patients who were offered rectal screening.

### Sample size

Our sample consisted of patients with a medical visit to the three participating PCPs during the observation period who self-identified as MSM or transgender female, were due for rectal screening, and opted for self-collection versus clinician-collection.

### Analysis

Descriptive statistics were analyzed using SPSS version 27 (Armonk, NY). Qualitative data on patient questions were captured and thematic content analysis was conducted using NVivo version 12 (QSR International, Melbourne, Australia). The study was approved by the Institutional Review Board at CHCI.

## Results

### Participant flow

**Fig. 1:**
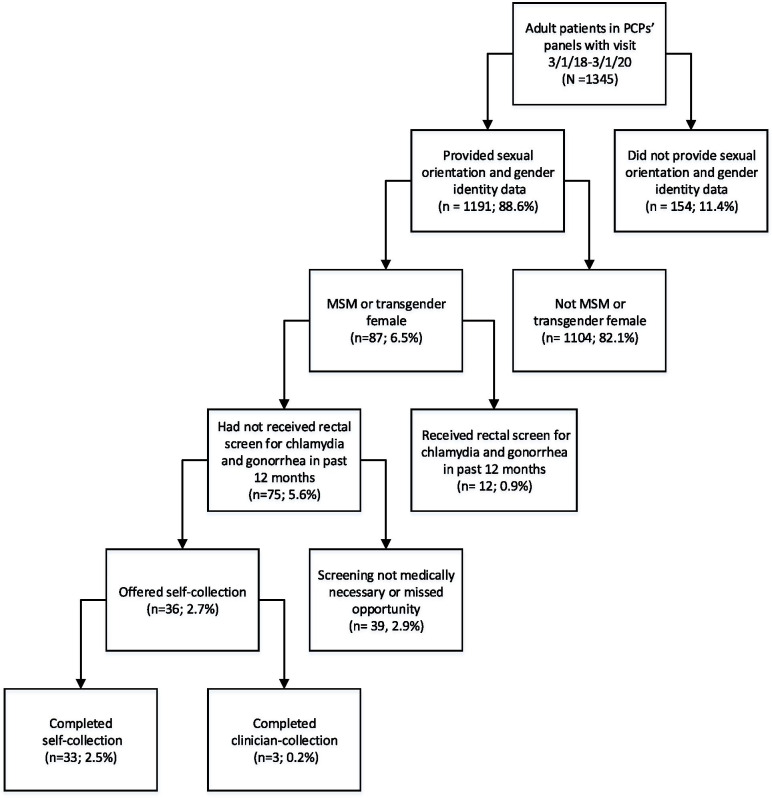
Eligibility screening. Of the 1345 unique patients seen by the 3 participating primary care providers (PCPs), 75 were men who have sex with men (MSM) or transgender females who were eligible for routine rectal screening, and 33 completed study participation. Percentages are reported out of a total of 1345 patients in the 3 PCPs’ panels

Figure 1 shows the flow of eligible participants through the study.

### Recruitment

Recruitment was initiated on March 1, 2018, following implementation of a Health Resources and Services Administration (HRSA)-mandated process to routinely collect and report SOGI data at FQHCs [22] and CHCI’s participation in a quality improvement initiative aimed at further increasing ability to use SOGI data to offer routine risk-based STI screening [23]. Recruitment was opportunistic and ended on March 1, 2020, due to restrictions on in-person visits imposed by the COVID-19 pandemic. The final sample (*n* = 33) represented 37.9% of MSM and transgender women who were seen by the participating PCPs for a medical visit (*n* = 87). The sample of patients who self-collected were similar to the general population of MSM and transgender women seen during the study period with respect to age, race, ethnicity, and self-reported sexual orientation and gender identity, and thus deemed sufficient to draw conclusions on the potential feasibility of rectal self-collection adoption.

### Numbers analyzed

Approximately 6.5% (*n* = 87) of the 1345 patients who presented for care during the study period identified as MSM (*n* = 67) or transgender female (*n* = 20) based on SOGI information from EHR data, and were potentially eligible for inclusion. Of the 87 potentially eligible patients, twelve (13.8%) were identified as having had a rectal STI screen within the past year, and were not due for screening on the visit date. Thirty-nine patients had screening deferred on the visit date (e.g., deemed not medically necessary or was a missed opportunity). Thirty-six patients who required and agreed to rectal screening were offered participation in the self-collection study. Three of these patients (8.3%) opted for clinician-collection due to current or prior history of rectal symptoms. All other patients (*n* = 33, 91.7%) chose self-collection (Fig. 1).

### Baseline data

Table 1 shows baseline data on the 33 patients who completed self-collection. All 33 were assigned male sex at birth (*n* = 33; 100%) with 26 identifying their gender as male (78.8%), five as transgender female (15.2%), and two chose not to disclose (6.0%). Participants were 39.4% White, 18.2% Black, 39.4% Hispanic/Latinx, and 3.0% other race/ethnicity, with mean age 40.0 (range, 19‑59; SD, 10.3). Most identified their sexual orientation as gay (*n* = 23, 69.7%) (Table 1).

**Table 1:** Patient demographics (*n* = 33)

	Self-collection (*n* = 33)
	*n* (%)
**Age**	
18‑29	6 (18.2)
30‑39	10 (30.3)
40‑49	12 (36.4)
50‑59	5 (15.2)
60+	0 (0.0)
**Race**	
Non-Hispanic White	13 (39.4)
Non-Hispanic Black or African American	6 (18.2)
Hispanic/Latinx	13 (39.4)
Other	1 (3.0)
**Sexual orientation**	
Straight or heterosexual	2 (6.1)
Gay or homosexual	23 (69.7)
Bisexual	2 (6.1)
Choose not to disclose	3 (9.1)
Other	3 (9.1)
**Gender identity**	
Male	26 (78.8)
Female or transgender female	5 (15.2)
Genderqueer	0 (0.0)
Choose not to disclose	2 (6.1)
Other	0 (0.0)

### Outcomes and estimation

#### Primary outcomes

*PCPs’ acceptability of self-collection*

*Time and effort.* All PCPs (*n* = 3) agreed that self-collection was time-efficient and not disruptive to the clinical workflow.

*Likelihood of rectal screening*. Two PCPs agreed and one somewhat agreed that self-collection increased their likelihood to obtain a rectal specimen from a patient.

*Preference.* Two of the three PCPs preferred patient self-collection to clinician-collection, and the third expressed no preference.

*Patients’ acceptability of self-collection*

*Ability to self-collect.* All 33 study participants endorsed comfort with self-collection and the majority agreed self-collection was easy (*n* = 31; 93.9%). The most common self-collection questions patients asked were as follows: (1) how to seal the swab into the tube after collection; (2) how far into the rectum to insert the swab; and (3) how much to move the swab once inserted rectally. Nearly all self-collected specimens (32/33; 97.0%) were suitable for laboratory analysis.

*Physical comfort.* The majority of patients (*n* = 27; 81.8%) were neutral, agreed, or strongly agreed that they did not feel pain during self-collection.

*Preference*. Two-thirds of participants preferred self-collection over clinician-collection (*n* = 23; 69.7%), and the remaining third expressed no preference (*n* = 10; 30.3%).

Please refer to Table 2 for details of PCP and patient surveys.

**Table 2:** Patient and PCP self-collection survey results

	Agree, 5.0	Somewhat agree, 4.0	Neutral, 3.0	Somewhat disagree, 2.0	Disagree, 1.0	Average score
*Patient survey* (*n =* 33)	*n* (%)	*n* (%)	*n* (%)	*n* (%)	*n* (%)	
The instructions were easy to follow	31 (93.9)	2 (6.1)	0 (0.0)	0 (0.0)	0 (0.0)	4.94
It was easy to swab my own bottom	27 (81.8)	4 (12.1)	2 (6.1)	0 (0.0)	0 (0.0)	4.76
I felt comfortable swabbing my own bottom	31 (93.9)	2 (6.1)	0 (0.0)	0 (0.0)	0 (0.0)	4.94
I did not feel pain when swabbing my own bottom	22 (66.7)	1 (3.0)	4 (12.1)	5 (15.2)	1 (3.0)	4.15
I felt I was able to ask questions about swabbing my own bottom	32 (97.0)	1 (3.0)	0 (0.0)	0 (0.0)	0 (0.0)	4.97
I prefer to swab my own bottom (vs. no preference or clinician-collection)^a^	23 (69.7)	N/A	10 (30.3)	N/A	0 (0.0)	4.39
*PCP survey* (*n =* 3)	*n* (%)	*n* (%)	*n* (%)	*n* (%)	*n* (%)	
Explaining the rectal swab self-collection procedure to the patient was easy	3 (100.0)	0 (0.0)	0 (0.0)	0 (0.0)	0 (0.0)	5.00
Patient rectal swab collection was more time-efficient than provider collection	3 (100.0)	0 (0.0)	0 (0.0)	0 (0.0)	0 (0.0)	5.00
Patient rectal swab collection was less disruptive to the clinical visit compared to provider collection	3 (100.0)	0 (0.0)	0 (0.0)	0 (0.0)	0 (0.0)	5.00
Patient self-collection increases the likelihood that I would collect a rectal swab during a clinical visit	2 (66.7)	1 (33.3)	0 (0.0)	0 (0.0)	0 (0.0)	4.67
Given a choice, I prefer that patients collect their own rectal swabs (vs. no preference or clinician-collection)^a^	2 (66.7)	N/A	1 (33.3)	N/A	0 (0.0)	4.33

Patients and primary care providers (PCPs) completed brief cross-sectional surveys to indicate their perceptions of rectal self-collection. All items were assessed on a 5-point Likert scale from “Disagree” (1.0) to “Agree” (5.0)

^a^Three choices were given: “Prefer patient self-collection” (5.0), “No preference” (3.0), “Prefer clinician-collection” (1.0)

#### Secondary outcomes

*Ability to identify patients for rectal self-collection*

The vast majority of patients (88.6%, *n* = 1191) had SOGI data documented in the EHR, and 87 identified as MSM or transgender female. Twelve of these patients would have qualified for routine rectal screening based on SOGI and sexual risk, but had already been screened within the prior year. Over half of the remaining 75 MSM and transgender female patients (*n* = 39) were not offered screening (Fig. 1). Potential reasons for non-screening on the date of the visit, such as patient refusal, lack of need based on sexual behavior, and missed opportunity were not uniformly documented and thus were unable to be analyzed.

## Discussion

### Limitations

Limitations of our study include having a small sample and lack of a clinician-collection comparison group. We relied on qualitative self-report of time savings from PCPs but did not collect data from MAs. Furthermore, we did not conduct a time study to collect precise data on time spent by PCPs or by MAs to administer self-collection. Our future study intends to examine the time spent and whether self-collection presents significant additional workload for non-PCP members of the primary care team. We were not able to capture specific rationale for non-screening for 39 patients who were MSM or transgender female and who had not completed rectal screening within the past 12 months. Our future study will capture these data in a structured field in the EHR, which will allow us to examine reasons for non-screening. While our health center collected SOGI data in the EHR, meaningful use of this information may not have been uniformly leveraged. Our future study will incorporate SOGI information into the clinical decision support tool MAs use for preventive screenings in order to identify MSM and transgender women who may require STI screening. Although we collected SOGI data, we did not yet have a standardized sexual risk assessment tool in our EHR. We plan to incorporate sexual risk assessment data in our future study and we encourage other health centers that anticipate initiating SOGI data collection to assess how SOGI and sexual risk assessment data in structured EHR fields could be used to improve STI screening, reporting, and analysis.

While self-collection can be used as a tool to decrease stigma associated with STI screening, patients may nevertheless feel stigmatized if self-collection is used disparately based on gender identity, sexual orientation, and anatomical site. Our future study will implement self-collection for urine, vaginal, and rectal specimens for all patients when shifting the task of STI screening to non-provider clinical members to align with other existing screening processes (e.g., urinalysis, hemoglobin A1C, blood sugar level, pregnancy test, toxicology screen) and will examine whether participants perceive any stigma when asked to self-collect their specimen.

We are aware that there may be a power dynamic that might influence a patient’s response to survey questions administered by members of the clinical team. Future studies should attempt to use non-clinical team members, preferably with lived experience, to administer surveys on perception of self-collection to all patients undergoing STI screening.

### Generalizability

Our feasibility study was conducted in a U.S. state and at a medical practice that provided above-average acceptance, support, and affirmation of sexual and gender minority people, which may limit generalizability of these findings to other settings where patients may feel less accepted and potentially uncomfortable disclosing sexual behaviors with their primary care team. The study was also concentrated at a primary care FQHC that collected SOGI data routinely. Such collection may help identify MSM and transgender women for potential screening more readily than at a practice that did not ask about SOGI information.

### Interpretation

This study assessed provider and patient acceptability of rectal self-collection and feasibility of offering it during a medical visit. We found that incorporating self-collection for rectal chlamydia and gonorrhea screening among MSM and transgender women was efficient, highly acceptable, and preferred by patients and clinicians. Our findings are in line with other studies indicating that patients prefer self-collection [17, 18] and provide additional insight into how to integrate it into routine primary care at a large FQHC serving the general population. We found that self-collection was just as effective as clinician-collection in producing a sample appropriate for laboratory analysis. The percentage of self-collected specimens that were not suitable for laboratory analysis (1/33 = 3.0%) was comparable to the percentage of all clinician-collected rectal specimens organization-wide between March 2019 and March 2020 that were not suitable for analysis (13/149 = 8.7%). While prior studies of rectal self-collection have assessed feasibility from a cost-effectiveness or specimen collection accuracy perspective [19, 24, 25], our study is unique in that we included patient-provided responses in our assessment of feasibility. Our findings also contribute to the body of qualitative knowledge on patients’ perception of pain or discomfort and ease of compliance with rectal self-collection procedure [26, 27] and corroborate what is known about the acceptability of the self-collection process [17]. Self-collection in FQHCs, which care for patients in the healthcare safety net, many of whom are racial and ethnic minorities who are disproportionately affected by STIs relative to non-Hispanic White patients [1], is a potential strategy to overcome access barriers.

Neglecting extra-genital testing in MSM and transgender women leads to missed diagnoses and can contribute to persistent disparities in STIs among sexual and gender minorities. Rectal mucosa is vulnerable to STIs and symptomatic or asymptomatic extra-genital chlamydia and gonorrhea infections are associated with increased risk of HIV transmission among MSM [7, 13, 28]. Identifying rectal infections, especially those without symptoms, provides an opportunity to discuss HIV risk and offer prevention strategies, such as HIV pre-exposure prophylaxis (PrEP). Since MSM and transgender women, especially those of color, may not have access to LGBT-focused health centers and are the most vulnerable to acquiring HIV, offering rectal self-collection in FQHCs may be the first critical step to STI and HIV prevention [1, 13].

The Centers for Disease Control and Prevention’s Recommendations for Providing Quality Sexually Transmitted Disease Clinical Services (2020) note that STIs are increasingly treated in primary care [29] and recommend that basic STI services should be made available in these settings. However, primary care community health centers, including FQHCs, have only recently (January 2018) been required to collect SOGI data [30], and often do not conduct risk-based sexual health screening [23]. Though our study demonstrates the feasibility of offering rectal STI screening during a primary care visit, further studies are needed to determine how primary care clinics can increase their capacity to offer comprehensive STI clinical services including sexual risk assessment, risk reduction counseling, and partner-services, which are commonly available in specialty STI clinics [31].

Nevertheless, continued emphasis on SOGI data collection in primary care health centers is critical. SOGI information must be recorded in the EHR in a systematic manner that allows for effective patient-level use by clinical teams and organization-level use for population health. The majority of our sample (*n* = 1191, 88.6%) had SOGI data on file. We found that 7.3% (*n* = 87) of these patients self-identified as MSM or transgender female, which exceeds the estimated percentage of U.S. adults who self-report any sexual and gender minority identity (4.5%) [32]. This illustrates the crucial role that FQHCs can play in tackling the STI burden that many members of sexual and gender minority populations carry, and which fuels the STI epidemic.

Despite the availability of SOGI information in the EHR, 39 patients potentially eligible for rectal screening did not receive it. Clinical decision support tools such as dashboards using SOGI data can thus identify patients with potential risks for STIs and consequently trigger any member of the clinical team to offer rectal self-collection, standardize and normalize STI screening, and provide opportunities to offer PrEP and maximize HIV prevention efforts. As this study demonstrated, MAs were able to facilitate patient rectal self-collection. Since nurses and MAs are already able to collect pharyngeal and urine specimens, rectal self-collection can shift the burden of STI testing from PCPs to clinical support staff by extending capability for MAs to assist with screening and for nurses to conduct comprehensive assessment and screening under standing orders from a PCP.

Our future study will implement STI services at this large FQHC that will leverage SOGI data and standardized sexual risk assessments, utilize a clinical decision support tool for MAs and nurses (the organization’s planned care dashboard), and set up templates and standardized protocols for nurse-led STI visits. Care team members will receive training on comprehensive STI screening that will permit them to work collaboratively to assess and respond to patients’ needs based on SOGI and sexual risk. Improved documentation of sexual risk in structured data fields in the patient’s chart is needed in order to accurately track screening rates among patients for whom it is medically indicated (i.e., not screened in the past 12 months, patient self-reported receptive sexual intercourse). We anticipate the ability to make this change in our practice as part of implementing a larger study.

STI rates continue to rise in the USA, disproportionately affecting certain populations like MSM and transgender women, particularly those of racial and ethnic minorities. As access to STI clinics becomes more limited, primary care centers, especially those catering to the medically underserved, need to ensure they offer comprehensive STI services. Rectal self-collection can and should be considered as part of any strategy to increase STI screening rates given that rectal specimen collection is the most invasive, discomforting, and time-consuming of all STI testing required for MSM and transgender women. Our study indicated that rectal self-collection was highly accepted and preferred by both PCPs and patients and was easily implemented in a busy safety-net primary care practice. This is a positive indication supporting use of rectal self-collection as a core component of our future study that will assess implementation of comprehensive STI services, leveraging the whole primary care clinical team including MAs and nurses, in a large FQHC setting.

## Data Availability

The datasets generated during and/or analyzed during the current study are not publicly available due to desire to protect the confidentiality of individual patients who identify as members of the minority sexual orientation, gender identity, and sexual risk behavior groups studied. Data are available from the corresponding author on reasonable request.
